# Use of hyperlinks in electronic test result communication: a survey study in general practice

**DOI:** 10.1186/1472-6947-12-114

**Published:** 2012-10-04

**Authors:** Thomas Ostersen Mukai, Flemming Bro, Morten Fenger-Grøn, Frede Olesen, Peter Vedsted

**Affiliations:** 1 The Research Unit for General Practice, School of Public Health, Aarhus University, Bartholins Allé 2, DK-8000, Aarhus C, Denmark; 2 Section for General Medical Practice, School of Public Health, Aarhus University, Bartholins Allé 2, DK-8000, Aarhus C, Denmark; 3 Department of Clinical Epidemiology, Aarhus University Hospital, Olof Palmes Allé 43-45, DK-8200, Aarhus N, Denmark; 4 Research Centre for Cancer Diagnosis in Primary Care – CaP, Aarhus University, Bartholins Allé 2, 8000, Aarhus C, Denmark

## Abstract

**Background:**

Information is essential in healthcare. Recording, handling and sharing healthcare information is important in order to ensure high quality of delivered healthcare. Information and communication technology (ICT) may be a valuable tool for handling these challenges. One way of enhancing the exchange of information could be to establish a link between patient-specific and general information sent to the general practitioner (GP). The aim of the present paper is to study GPs' use of a hyperlink inserted into electronic test result communication.

**Methods:**

We inserted a hyperlink into the electronic test result communication sent to the patients’ GPs who participated in a regional, systematic breast cancer screening program. The hyperlink target was a web-site with information on the breast cancer screening program and breast cancer in general. Different strategies were used to increase the GPs’ use of this hyperlink. The outcome measure was the GPs’ self-reported use of the link. Data were collected by means of a one-page paper-based questionnaire.

**Results:**

The response rate was 73% (n=242). In total, 108 (45%) of the GPs reported to have used the link. In all, 22% (n=53) of the GPs used the web-address from a paper letter and 37% (n=89) used the hyperlink in the electronic test result communication (*Δ* = 15*%*[95*%confidence*  int *erval*(*CI*) = 8 − 22*%P* < 0.001]). We found no statistically significant associations between use of the web-address/hyperlink and the GP’s gender, age, or attitude towards mammography screening.

**Conclusions:**

The results suggest that hyperlinks in electronic test result communication could be a feasible strategy for combining and sharing different types of healthcare information.

## Background

Information can be categorised according to its level of privacy [1]. Boyne's model consists of three categories or levels: Level C: information relevant to all humans, often found in theoretical exposition like in textbooks. Level B: information relevant to on a subcategory of humans, e.g. patients with breast cancer; often communicated via scientific articles and, increasingly, via the Internet. Level A: information relevant to on a specific patient, e.g. the results of mammogram; often kept in healthcare record systems. Hyperlinks may be used to link information that belongs together because it addresses the same topic, but which is separated for reasons of privacy.

Information is essential in healthcare. Recording, handling and sharing healthcare information is important in order to ensure high quality of delivered healthcare [2]. Some diseases and especially cancer pathways are complicated trajectories that involve multiple actors. This calls for continuously updated information and well-functioning communication channels to ensure due coordination, integration, effectiveness and shared decision-making. In Denmark, the general practitioner (GP) plays an important role in the patients' cancer pathways [3], not least because the GP serves as a gatekeeper to specialised healthcare. About 98% of Danish citizens are listed with a specific GP[4].

Information and communication technology (ICT) is often used for communication and information exchange purposes [5]. This purpose may be served by deploying a centralized strategy that resorts to web-based ICT; or by deploying a decentralized strategy that makes use of software installed in each healthcare provider's local setting. Contrary to web-based resources, locally installed ICT tools require GP resources for installation and maintenance [6]. On the other hand, the current information overload on the Internet makes web-based solutions a less attractive option than locally installed tools [7].

Danish GPs have been using electronic medical records for decades and electronic record coverage has now reached 100% [8,9]. The Danish non-profit organisation MEDCOM has developed nation-wide standards for the most frequent cross-sector communication flows in the health care sector [10]. These standards are based on the United Nations Electronic Data Interchange for Administration, Commerce and Transport (EDIFACT) standard [11]. A secured network, the Healthcare Data Network, connects providers in primary and secondary care and enables confidential and encrypted data exchange.

After every in- or out-patient contact with a hospital an electronic discharge summary letter is sent to the patient’s GP. Furthermore, when a GP orders an x-ray, blood tests or microbial analysis, the results are automatically returned electronically to the GP. Such discharge letters and test result communication (Level A information) enable the GP to coordinate further care.

The present paper hypothesizes that using hyperlinks inserted into the electronic communication (Level A information) may be a useful method for increasing access to existing, patient-oriented information with general information about the disease and its treatment via the web (Level B information).

Hence, the aim was to test this hypothesis by examining to which extent a hyperlink inserted into mammography test result communication to the GPs is actually being utilized by the GPs. First, we compared the use of web-addresses in paper information letters with the use of hyperlinks in electronic test result communication. Second, we investigated whether the GP’s gender, age or attitude towards mammography screening modified his or her use of the hyperlink. Finally, we analysed if priming the GPs with e-mails enhanced their use of hyperlinks.

## Methods

### Setting

A nationwide breast cancer screening program was introduced in Denmark in 2008. The program was enacted to offer women between 50 and 69 years of age a systematic screening by mammography biannually. The Department for Public Health Programs in The Central Denmark Region (1.2 million citizens) invited all women attending a given GP clinic to join the program.

The program affords the GP a key role as the one who informs the patient about her possibility to participate and who provides follow-up information if needed [12]. These key roles makes it of outmost importance that the GP has access to the best possible specific information about the individual patient as well as general information about the screening program. The Department issued leaflets describing every step in the screening pathway. A group of GPs confirmed the relevance to the GPs of the contents of these leaflets. Concurrently, a project web page (http://www.mammografiscreening.dk) was established featuring information identical to that contained in the leaflet. The target group of the web page was the health professionals, mainly the GPs.

**Figure 1:**
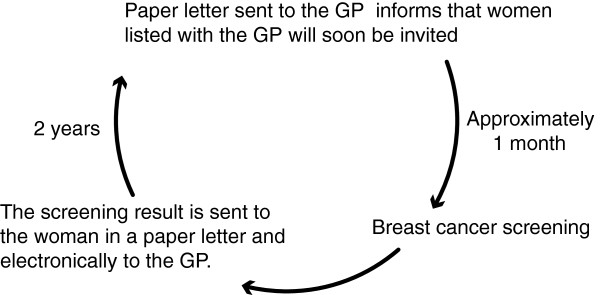
Graphic overview of the breast cancer screening program.

The breast cancer screening program was organised so that all women listed with the same GP or the same practice were invited in the same period. The organising department estimated that the average period for women on the same list to complete the program was one month. Approximately one month before the first women on their list were scheduled for screening, the GPs received a paper letter from the booking secretariat informing them GP about the program. The information leaflets were attached to this letter. After each mammography, the test result was sent directly to the woman and electronically to her GP (Figure 1).

### Intervention

**Figure 2:**
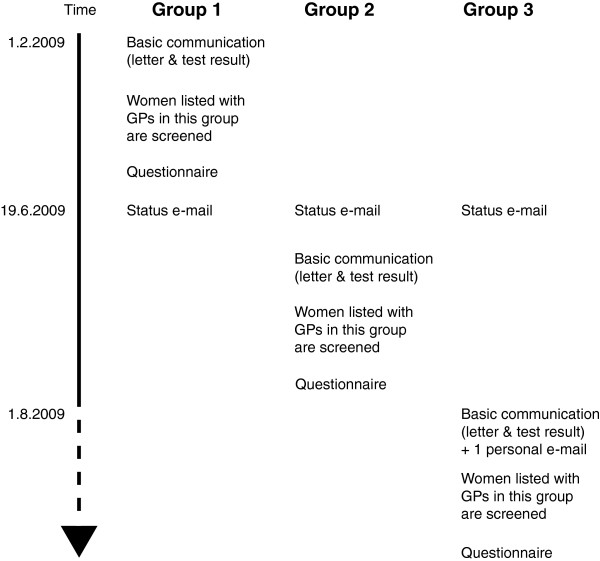
Graphic overview of the timing of the study.

The intervention consisted of inserting a hyperlink to the project web page into the electronic test result communication. This hyperlink granted the GP a one-click access to the target web page. From 1 February 2009, the booking secretariat began to insert the project web page address into the initial paper letter and the automatic insertion of hyperlink into the test result communication was activated. From this point in time, all GPs received the basic communication consisting of a web-address/hyperlink in an initial paper letter and the electronic test result communication, respectively. By 19 June 2009, the booking secretariat sent a status e-mail to all GPs in the Region to inform them about the screening project. We inserted a hyperlink into this e-mail. From 1 August 2009, the booking secretariat sent a personal e-mail to each GP two days after they had sent the initial paper letter. The contents of this e-mail were identical to that of the paper letter except that the web-address was replaced with a hyperlink to the project web page. A graphical overview of the study design and its flow can be found in Figure 2.

We consecutively included a total of 300 GPs from 148 clinics. The GPs were divided into three groups based on the order of the invitation list made by the booking secretariat from 1 February 2009 to 31 October 2009. The groups were exposed to the intervention in various ways:

Group 1 (n=100) had the usual communication consisting of a paper letter with the web-address and the electronic test result communication containing the hyperlink.Group 2 (n=100) had the usual communication plus one e-mail (the status e-mail), containing the hyperlink.Group 3 (n=100) had the usual communication plus two e-mails (both the status and the personal e-mail) containing the hyperlink.

### Outcome measure

Our outcome measure was the GP’s self-reported use of the hyperlink. Data were collected with an ad-hoc-developed one-page, paper-based questionnaire. We pilot tested the questionnaire among 30 GPs. Since all pilot testing GPs received the usual communication and because we did not alter the questionnaire after the pilot test, we included the pilot testing GPs in group 1.

The questionnaire was sent to the GPs approximately two months after the initial letter had been sent by the booking secretariat (Figure 2). Non-responding GPs were sent a reminder after two weeks. Participation was voluntary and none of the GPs were compensated in any way for their time use.

During the study, we also recorded the web pages' visiting statistics in term of unique visitors.

### Data

Items regarding use of the link were dichotomised into users (answered "Yes") and non-users (answers "No", "Don't know" and "Hasn't received"). Questionnaires were scanned and verified using the Cardiff Teleform software.

The unique visitors were identified based on their internet-protocol (IP) addresses and registered on a daily basis. Furthermore, we were able to tell whether the web page was accessed via the hyperlink in the status e-mail, via the personal e-mail or via letter/electronic test result.

### Analysis

McNemar's test of paired binary data was used for testing differences between proportions. The cohort study risk ratio (RR) was used to estimate the effect of priming with e-mails. Logistic regression was applied for estimating and testing odds ratios (OR) for the univariate model of association between the GP’s gender, age and attitude towards mammography screening and the GP’s use of the hyperlink.A 5% significance level was chosen. Stata v.11 was used for all statistics.

### Approvals

According to the Scientific Ethics Committee in the Central Denmark Region, this study did not need approval by the Committee. The study was recommended by the Multi Practice Committee of the Danish Society of General Practitioners and the Association of Danish General Practitioners (MPU 04–2009).

## Results

The survey had a response rate of 73% (n=242); 63% were males and their mean age was 54 years (36–70 years) (Table 1). Responding female GPs were 3.4 [*CI* = 1.4 − 5.3](*P* = 0.0007) years younger than responding male GPs. Among non-responding GPs, 70% were males. All GPs in this study, except one, encountered at least one woman with a positive finding at the mammography screening.

**Table 1:** Survey results

		**Group 1**		**Group 2**		**Group 3**		**Total**	
		**n**	**%**	**n**	**%**	**n**	**%**	**n**	**%**
**Questionnaires sent**		130		100		100		330	
**Questionnaires received**		91	70.0	79	79.0	72	72.0	242	73.3
**Gender:**									
	Female GP	38	41.8	26	32.9	25	34.7	89	36.8
	Male GP	53	58.2	53	67.1	47	65.3	153	63.2
**Attitude towards mammography screening:**									
	Positive	71	80.7	61	79.2	51	75.0	183	78.5
	Negative	10	11.4	8	10.4	3	4.4	21	9.0
	Undecided	7	8.0	8	10.4	14	20.6	29	12.5
**Used link:**									
	In letter	17	18.7	21	26.6	15	20.8	53	21.9
	In e-mail	-	-	22	27.9	24	33.3	46	19.0
	In test result	27	29.7	34	43.0	28	38.9	89	36.8
	At all	36	39.6	40	50.6	32	44.4	108	44.6
**Mean age (range)**		52.6	(36–69)	53.0	(38–70)	55.3	(36–69)	53.5	(36–70)

A total of 108 (45%) GPs reported to have used the link (Table 1). We found no difference between the use of hyperlinks in paper letters (22%, n=53) and e-mails (19%, n=46) (P=0.318). In all, 37% (n=89) had used the hyperlink in the electronic test result communication, which was significantly more than had used the link included in paper letters (*Δ* = 15*%*[*CI* = 8 − 22*%*](*P* < 0.001)). The difference between use of hyperlinks in electronic test result communication and e-mails was 18% ([*CI* = 11 − 25*%*](*P* < 0.001)). Some GPs (14%, n=34) used the hyperlink in both the paper letter and the electronic test result communication.

If GPs had been primed with an e-mail, they were more likely to use the hyperlinks in the test results, but the difference was not statistically significant (RR=1.38; [CI= 0.96 to 2.00] p=0.0751). We found no significant association between the number of priming e-mails and the GP’s use of the hyperlink in the test result (Table 2).

**Table 2:** GPs’ use of the hyperlink in the electronic test result communication in relation to receiving a priming e-mail

**Used hyperlink in test results**	**Received a priming e-mail**				**Total**
	**Yes**		**No**		
Yes	62	(42%)	27	(30%)	89
No	89	(58%)	64	(70%)	153
Total	151	(100%)	91	(100%)	242

There were no statistically significant associations between the GP’s use of the web-address/hyperlink and the GP’s gender, age and attitude towards mammography screenings. However, if a GP was negative towards breast cancer screening, there was a tendency for the GP not to use the hyperlink (Table 3).

**Table 3:** Odds ratios (OR) for GPs’ use of web-address in letters, hyperlinks in electronic test results and in total

	**Letter**			**Electronic test result**			**Used link at all**		
	**OR**	**95% CI**	**p**	**OR**	**95% CI**	**p**	**OR**	**95% CI**	**p**
**Gender**									
Female	1			1			1		
Male	1.30	0.70;2.41	0.411	1.25	0.68;2.31	0.477	1.06	0.62;1.80	0.835
**Age group**									
≥50	1			1			1		
<50	1.27	0.66;2.42	0.476	0.92	0.51;1.65	0.772	0.9	0.49;1.62	0.716
**Attitude towards mammography screening**									
Positive	1			1			1		
Negative	-*	-*	-*	0.51	0.15;1.69	0.269	0.34	0.10;1.17	0.086
Not decided yet	0.82	0.32;2.13	0.641	0.88	0.37;2.09	0.777	0.99	0.45;2.18	0.972

*The missing results arise because no GPs with a negative attitude towards mammography screening used the web-address from the paper letter.

During the study period, the front pages of the web page were the most frequently visited of all the web pages (Table 4). The web pages that were linked to from the personal e-mail were visited nearly twice as much as the web pages from the first, general e-mail.

**Table 4:** Monthly visiting rates for unique visitors on the web pages linked to by the hyperlink in 2009

		**Title of the sections on the web page**						
	**Month**	**Breast cancer screening in general (Front page)**	**Breast cancer in general**	**Invitation of the women**	**Description of breast cancer screening**	**Description of clinical breast cancer screening**	**Description of surgical treatment**	**Description of adjuvant treatment**
Access via	Feb	107	18	28	22	16	10	6
web address / hyperlink	Mar	88	9	33	15	11	3	0
in letter / Test result	Apr	68	20	4	4	4	4	2
	May	112	16	33	18	11	11	5
	June	125	18	38	15	14	14	7
	July	60	5	15	11	8	2	1
	Aug	81	26	24	12	10	2	1
	Sep	75	5	24	9	7	2	2
	Oct	77	10	16	12	9	2	1
	**Total**	**793**	**127**	**215**	**118**	**90**	**50**	**25**
Access via hyperlink in status e-mail	July	38	8	11	2	3	3	4
	Aug	12	8	9	3	11	3	3
	Sep	20	3	10	10	8	8	13
	Oct	5	4	2	2	7	1	6
	**Total**	**75**	**23**	**32**	**17**	**29**	**15**	**26**
Access via hyperlink in personal e-mail	July	6	11	7	3	10	5	2
	Aug	55	17	17	12	20	9	4
	Sep	54	16	21	18	19	4	4
	Oct	34	25	14	9	13	5	5
	**Total**	**149**	**69**	**59**	**42**	**62**	**23**	**15**

**Table 5:** Non-standardised translation of the Danish questionnaire

**What is your gender?**	**Female**	**Male**			
**How old are you?**					
**Which electronic medical record system do you use?**	Profdoc Æskulap	MedWin	Profdoc Darwin	Novax	
	Ganglion	Profdoc MediCare	PC-praksis	EMAR	
	PLC, A-Data	MyClinic	MultiMed	Docbase	
	No system		Other system		
**Which of following statements do most accurately describe your attitude towards breast cancer screening?**	I am mostly positive towards breast cancer screening	I am mostly negative towards breast cancer screening	I have not yet decided		
**How much have you been:**					
**Contacted by women with questions about breast cancer screeningbeforethe women in your clinic were invited?**	Not at all	A little	Some	Much	
**Contacted by women with questions about breast cancer screeningafterthe women in your clinic were invited?**	Not at all	A little	Some	Much	
**Contacted by women with questions about breast cancer screeningbeforethe women in your clinic were invited?**	Not at all	A little	Some	Much	
**Have you used the hyperlink inserted into:**					
**The paper letter you received prior to the breast cancer screening in your clinic?**	Yes	No	Don't know	Not received	
**The e-mail sent to you prior to the breast cancer screening in your clinic?**	Yes	No	Don't know	Not received	
**The electronic test result communication after the breast cancer screening**	Yes	No	Don't know	Not received	
**The electronic test result communication after the clinical breast cancer screening?**	Yes	No	Don't know	Not received	
**Did you reach the web page when you clicked the hyperlink in:**					
**The electronic test result communication after the breast cancer screening**	Yes	No	Don't know	Not received	
**The electronic test result communication after the clinical breast cancer screening?**	Yes	No	Don't know	Not received	
**How often did you click on the hyperlink?**	0	1-2	3-5	6-10	10+
**Did you feel that you were capable of guiding the women in relation to breast cancer screening?**	Yes	No	Don't know		
**Did you feel that you were capable of guiding the women about the coming trajectory after a positive finding?**	Yes	No	Don't know		
**Did the hyperlink affect your guiding in these matters?**	Yes	No	Don't know		
**Overall, do you find the idea of the hyperlink to be useful?**	Yes	No	Don't know		

## Discussion

### Main findings

We found that the GPs were using the hyperlink in the electronic test result communication more than the web-address contained in the paper letter or in an e-mail. We found a possible effect of priming with an e-mail, but the effect was not statistically significant; and no significant difference in the use of the hyperlink was seen between GPs primed with one or two e-mails. Gender, age or attitude towards mammography screening did not significantly influence the chance of a GP using the hyperlink.

We found no difference between paper letter and e-mail letter use of hyperlinks, and easy access via one-click did not in itself increase the use of the hyperlinks. It is likely that relevance and timeliness are more important than easy access as usage increased when the hyperlinks were inserted into patient-specific communication. In some respects, the hyperlink could be considered a reminder; in the present case, a reminder that gave access to additional information. A Cochrane review showed that reminder functionalities in general had small to modest effects and only a minority had large effects [13].

Priming the GP with an e-mail had a slight, positive effect on their use of the hyperlink in the electronic test result communication even if the effect was not statistically significant. The absence of a statistically significant effect may be due to low statistical precision, but until the results of a larger study are available, priming the GP with e-mail could still be beneficial, notably as the cost of sending an e-mail is negligent.

We found no statistically significant associations between the use of a link and GP factors like gender, age or attitude towards breast cancer screening. This makes us assume that the use of the hyperlink is not limited to a particular group, e.g. males or younger GPs, but that it is, indeed, widely used. Prior studies have described gender-dependent differences in use of ICT [14,15] in which women tend to use ICT to a lesser degree than men. We found no differences in hyperlink use between genders, which could be owing to the technological development in general and to the fact that confidence in using the internet and ICT has risen much since these prior studies were performed (1999 and 1998).

### Strength and weaknesses

This study included a large sample of GPs and women invited to attend a mammography screening. However, the confidence intervals in relation to the characteristics influencing the GPs’ use of hyperlinks were wide which implies that our statistical precision was not sufficiently high to detect small differences and this induces a risk of type II errors. Although the study was not designed as a randomised controlled trial, the pragmatic selection of the GPs took place at random using the chronology that guided the assignment of their patients. This minimizes the risk of bias. Also, our data stem from an intervention conducted in a real life situation under naturally occurring conditions and the findings can therefore be extrapolated to similar settings.

Another potential problem by the study design is that it makes it difficult to interpret the findings of the effect of priming the GPs with e-mails containing a hyperlink due to the timing of the e-mails and the questionnaires. This timing is important because it induces a risk of a prolonged exposure of the hyperlink. An overexposure will lead to an overestimation of the effect of priming with e-mails. Although we had planned the distribution of the hyperlink via e-mail before the project started, this part of the project ended up being more pragmatic than anticipated which impairs our ability to make inferences on the basis of the results of this part of the study significantly.

We used questionnaires to evaluate the use of hyperlinks and it is possible that those most likely to respond were the ones who reported to use of the web link, which would tend to overestimate the use of hyperlinks. Furthermore, it is possible that the GPs were unable to recall whether they had used the hyperlinks. Recall bias in the study could lead to both over- and under estimation of the use of hyperlink.

The web page statistics only provide a rough estimate of the use of the target web pages. By choosing to register unique visitors only and because the registration took place on a daily basis, we were not able to differentiate between the visitors. If a GP visited the web page every day, (s)he would count as a unique visitor every day. Opposite, multiple GPs, e.g. in a partnership practice, would have the same IP address and therefore only count as one unique visitor each day. Thus, we know that the web sites have been used, but we cannot determine the precise extent of the use.

Similarity between physicians in the same clinic may have given rise to a cluster effect and, hence, an overestimation of the difference. However, we consider the use of ICT and a hyperlink mainly to be the choice of the individual GP, and any cluster effect is therefore expected to be small.

Our study was designed to evaluate the use of hyperlinks and thus does not allow us to determine an effect of the offered information on the GPs' clinical behaviour, which should be investigated in future research.

## Conclusions

Hyperlinks in electronic test result communication are a feasible strategy for combining and sharing different categories of healthcare information between primary and secondary health care.

## Competing interests

The authors declare that they have no competing interests.

## Authors’ contributions

PV, FB, FO all conceived the study, participated in its design and helped to draft the manuscript. MFG helped with the statistical analysis as well as the drafting of the manuscript. TOM conceived the study, developed the intervention, conducted the survey, performed statistical analysis, and drafted the manuscript. All authors have read and approved the final manuscript.

## Pre-publication history

The pre-publication history for this paper can be accessed here:

http://www.biomedcentral.com/1472-6947/12/114/prepub
